# A systematic review of health economic studies on endotracheal tubes in preventing ventilator-associated pneumonia

**DOI:** 10.1097/MD.0000000000043877

**Published:** 2025-08-08

**Authors:** Benjamin De Vlam, Safeer Khan, Guy Hans

**Affiliations:** a University Hospital Antwerpen (UZA), Edegem, Belgium; b Department of Pharmaceutical Sciences, Institute of Chemical Sciences, Government College University, Lahore, Pakistan.

**Keywords:** endotracheal tube, health economic evaluation, subglottic suctions endotracheal tube, ventilator-associated pneumonia

## Abstract

**Background:**

Ventilator-associated pneumonia (VAP) places a substantial financial strain on both individuals and the healthcare system. There is ample evidence supporting the clinical utilization of different endotracheal tube (ETT) modifications to reduce the occurrence of VAP. However, there has been a lack of comprehensive assessment for their cost-effectiveness. This systematic review aims to thoroughly assess health economic studies that specifically examine the use of various modifications of ETTs in preventing VAP.

**Methods:**

An extensive search was conducted across 5 medical databases to find all full economic evaluations conducted between January 2010 and December 2023. The assessment of the studies’ quality was conducted using the updated Consolidated Health Economic Evaluation Reporting Standards statement.

**Results:**

A total of 4 economic assessments were identified, comprising of 3 studies of good quality and 1 study of medium quality, carried out in 3 different nations. The studies employed the Venner-PneuX (VPX) system ETT, the ETTs with heat and moisture exchangers filter, and the subglottic suction ETT. The comparator group consisted of either a standard ETT in 3 studies or multiple preventative strategies in 1 study. All 4 studies shown that the utilization of modified endotracheal ETTs holds significant promise in reducing the incidence of VAP from both a societal and hospital perspective. In particular, the utilization of ETT subglottic suctions was determined to be cost-effective in 2 studies.

**Conclusion:**

The use of modified ETT could result in improved patient outcomes and more efficiency in utilizing healthcare resources. Nevertheless, the result remains tentative due to the limited number of studies included and the heterogeneity in their methodology. Henceforth, it is important to carry out more extensive economic assessments by utilizing larger and more representative sample sizes.

## 1. Introduction

Ventilator-associated pneumonia (VAP) is the predominant hospital-acquired illness among patients undergoing mechanical ventilation.^[1]^ The incidence of this condition varies between 5% and 67%, depending on the case mix and the diagnostic criteria employed.^[2]^ On average, there are approximately 5 cases per 1000 ventilator days.^[1]^

VAP imposes a significant economic burden on both patients and the healthcare system. It is linked to prolonged stays in the intensive care unit (ICU) and hospital.^[1]^ Additionally, it necessitates extended periods of mechanical ventilation and increased respiratory support. Consequently, the average cost for treating patients with VAP amounts to £15,124, compared to £6295 for those without VAP. The additional expense associated with managing VAP patients is £8829.^[3]^ Similarly, patients suffering from comorbid conditions like chronic obstructive pulmonary disease, obesity, diabetes, and alcoholism represent a high-risk category for VAP,^[4]^ which in turn escalates the financial burden associated with these disorders. Additionally, complications arising from VAP, such as sepsis, significantly affect both the quality of life and the financial strain on healthcare systems with limited resources.^[5]^

The treatment of VAP is difficult because colonies grow in the airways, leading to changes in quorum sensing and the production of virulence factors. Therefore, both oral and systemic antibiotics face inherent obstacles in their efficacy, which are worsened when bacteria develop greater resistance.^[6]^ Consequently, the primary focus of the clinicians is on preventing it. There are several empirically validated strategies have been developed for the prevention of VAP.^[7]^ Within the realm of strategies, it is imperative to prevent, minimize, and combat the presence of pathogenic bacteria at the endotracheal tube (ETT) to mitigate this problem.^[6]^ These strategies involve the utilization of antiseptic solutions for oral hygiene, regular elimination of bacteria-laden subglottic secretions, and the implementation of specialized design and maintenance for the ETT.^[7]^

Novel ETT technologies have been devised to mitigate the occurrences of VAP. The subglottic secretion drainage-ETT cuff appears to be a reliable and cost-effective method for reducing VAP among patients.^[8]^ Various other technologies have been developed to improve the efficacy of ETT cuffs in preventing VAP. These innovations encompass the utilization of polyurethane to modify the material of the cuff, changing its shape to either cylindrical or tapered, and including pressure monitoring sensors to enhance the effectiveness of sealing. In addition, certain strategies have been employed to hinder the development of biofilms, including the utilization of methods such as silver coating, mucus shaver, and photodynamic therapy.^[9]^

There is abundant data supporting the clinical use of various ETT modifications to reduce the incidence of VAP.^[9]^ Nevertheless, up until now, there has been no thorough evaluation of the literature examining the economic outcomes of the use of different ETT in the ICU for the prevention of VAP. Hence, the comprehensive pattern of economic conclusions and the quality of the research that substantiates them remain ambiguous.

As health economic studies offer decision-makers valuable insights to optimize the allocation of resources and maximize health outcomes.^[10]^ Therefore, incorporating cost-effective techniques into routine clinical practice is essential to reduce the risk of VAP and minimize the accompanying financial burden. This approach not only mitigates VAP-related expenses but also ensures that patients receive the highest standard of care possible.^[11]^ This systematic review seeks to comprehensively evaluate health economic studies that explicitly investigate the utilization of different modifications of ETTs in the prevention of VAP. This will offer a comprehensive comprehension of their economic and clinical consequences in the prevention of VAP.

## 2. Methodology

### 2.1. Search strategy

A comprehensive examination of scientific literature was undertaken in accordance with the preferred reporting items for systematic reviews and meta-analyses standards.^[12]^ The search encompassed the timeframe spanning from January 2010 to December 2023, with the selection of the first year based solely on the relevance of updated data.

The literature review included 4 databases, that is, PubMed, ScienceDirect, Health Technology Assessment, and Cost-Effectiveness Analysis registry along with the search engine Google Scholar. Furthermore, a bibliographic search was performed on relevant journal articles, systematic reviews, and meta-analyses in the same databases.

The search terms employed focused on “health economics,” “ventilator-associated pneumonia,” and “endotracheal tubes.” Both free-text keywords and Medical Subject Headings terms were utilized to ensure comprehensive retrieval of pertinent studies. The search strategy was specifically designed to incorporate a variety of synonyms, abbreviations, and spelling variations, thereby capturing the diverse terminologies used across different studies. Boolean operators (AND, OR) and quotation marks (“.”) were also employed to refine and optimize the search results.

The search was conducted by 2 authors (SK and DVB) and any disagreements were resolved through discussion. Following the elimination of duplicates, titles, abstracts, and full-text articles underwent a comprehensive evaluation to remove research that did not match the inclusion criteria. The search strategy employed, and the number of retrieved results obtained are outlined in Supplementary material 01, Supplemental Digital Content, https://links.lww.com/MD/P647.

### 2.2. Selection of studies

To enhance the precision of the study selection criteria, we adhered to the population, intervention, comparator, outcome, and study design framework.

Population (P): The studies should include participants without any limitation on age, gender, and severity of underlying diseases, diagnostic criteria and comorbid conditions who are undergoing mechanical ventilation and are at risk of or have developed VAP.

Intervention (I): The study focuses on the use of customized ET tubes with the goal of avoiding VAP. Examples of such alterations may include tubes with specialized coatings, pressurized system, unique shapes, or built-in suction systems.

Comparison (C): Comparators should be comprised of standard ET that do not possess specific attributes aimed at reducing VAP, or other alternative methods for preventing VAP, or no comparison at all.

Outcomes (O): The primary outcomes of interest comprise the incidence of VAP, the cost-effectiveness of different types of endotracheal tubes, and any health economic outcomes such as duration of hospitalization, overall treatment expenses, and utilization of resources. Additional outcomes may include patient mortality, occurrence of comorbidities, and assessments of quality of life.

Study design (S): The included studies consist of randomized controlled trials and observational studies, all in English language and limited to those that conducted health economic evaluations, with a specific emphasis on cost-effectiveness and cost-utility studies. The exclusion criteria encompassed reviews, method or protocol papers, conference papers, case reports, editorials, letters, and correspondences.

### 2.3. Data extraction and quality appraisal

Each author participated in the review process, including study selection, quality assessment, and data extraction. The uniform data form was utilized to collect extensive information, encompassing the first author’s name, the study’s country and year, study design, sample size and demographics, type of underlying disease, specific details of the investigated ETT types, type of comparator, cost perspectives, currency, discount rates, relevant clinical outcomes, associated costs, health economic outcomes, limitations, conclusions, and funding sources.

The updated consolidated health economic evaluation reporting standards (CHEERS) statement was used to evaluate the quality of the provided economic studies. The updated CHEERS statement is designed to be adaptable to a wide range of health economic evaluations, incorporating novel methodologies and advancements in the area.^[13]^

The updated CHEERS checklist consists of 28 items that cover various aspects of economic evaluations. Each item was evaluated to see if a study completely met the standards, failed to meet the criteria, or was not relevant. Studies that attained a compliance rate of 100% were deemed to possess excellent quality. Likewise, studies that followed 75% to 99% of the criteria were categorized as high quality, but those that met 50% to 74% of the criteria were considered to have moderate quality. A compliance rate below 50% signifies poor quality.

### 2.4. Data synthesis

The data obtained from the studies were presented through a combination of narrative synthesis and organized tables. This technique is consistent with the recommendations for creating concise and informative summaries of health economic studies, as specified in the Cochrane Handbook for Systematic Reviews.^[14]^

## 3. Results

### 3.1. Literature search

**Figure 1.**
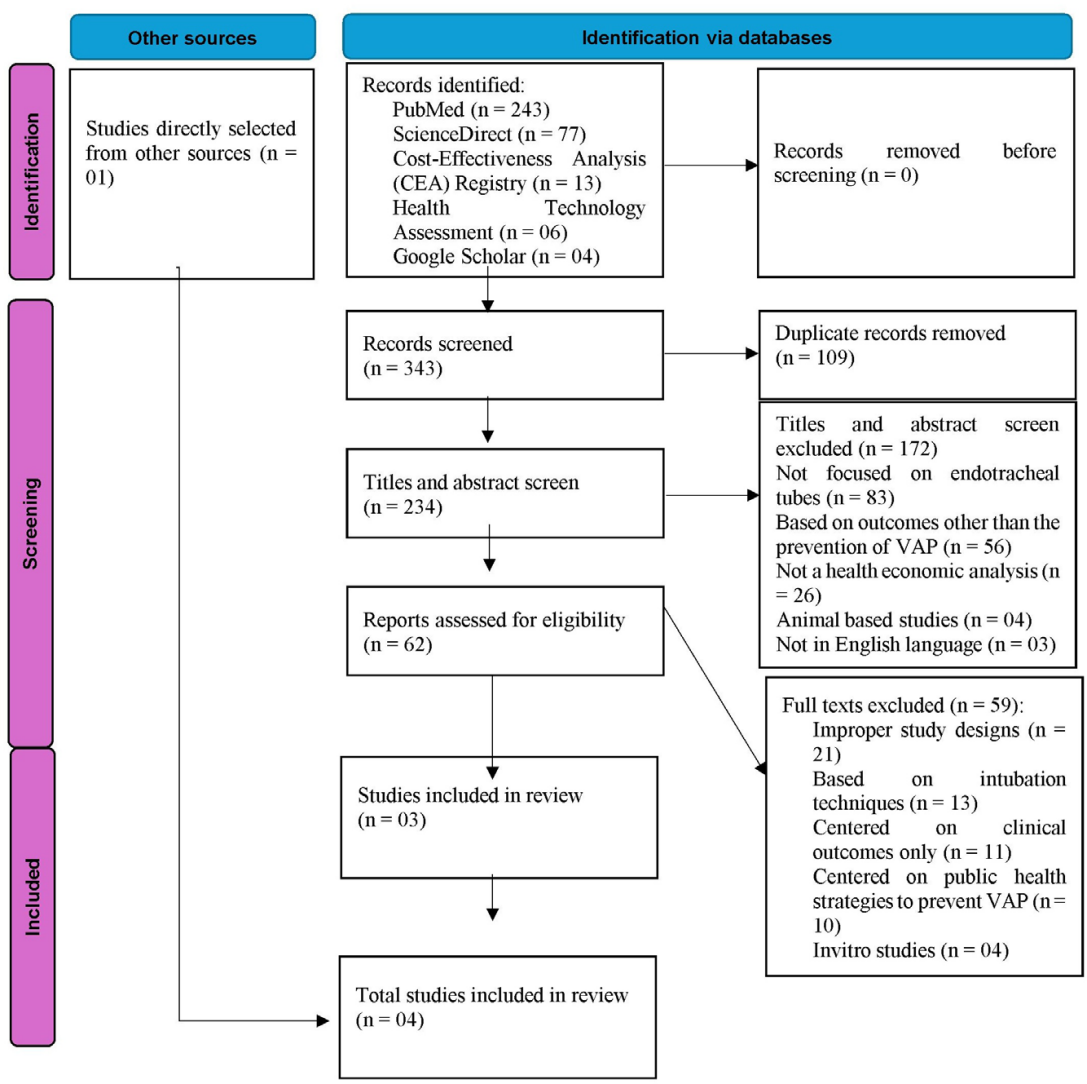
PRISMA flow chart of the searching and screening studies.

Upon performing the initial literature search, a total of 343 studies were initially identified. After eliminating 109 duplicate studies, 234 studies remained for further examination. After reviewing the titles and abstracts, we found 62 studies that met our inclusion criteria. The other 172 research were excluded for different reasons, as indicated in Figure 1. Among these, 59 studies were eliminated at the full-text stage due to various reasons, 21 studies had improper study designs, 13 studies based on intubation techniques, 11 studies centered only on clinical outcomes, 10 studies focused on public health strategies to prevent VAP, and 4 studies were based on in vitro investigations. Thus, there were 3 studies that satisfied the specified criteria for inclusion. In addition, one another study was retrieved directly from a relevant journal.^[15]^ A total of 4 studies were finally selected for the systematic review. The flow chart in Figure 1 illustrates the procedure of search and selection.

### 3.2. Quality assessment of included studies

According to the updated CHEERS assessment criteria, 3 out of 4 studies obtained ratings ranging from 75% to 99%, suggesting a high level of quality. A single study achieved a score of 68% and was categorized as possessing a moderate degree of quality.^[15]^

By utilizing the updated CHEERS tool statement of 2022,^[13]^ we found that none of the research included in our analysis met the majority of the newly included criteria. These components encompass the analysis of how the distribution of affects is characterized, the implementation of a strategy to involve patients and other affected individuals, and the evaluation of the consequences of interacting with patients and other affected individuals.

Several items in the selected studies were not relevant in the majority of studies. For example, because all of the studies had a limited time frame, the discount rate only applied to none of the investigations. Furthermore, neither of the individual studies performed a subgroup analysis, therefore rendering it unfeasible to apply the criteria for categorizing heterogeneity. The quality evaluation of the studies as per Updated CHEERS statement is provided in Table 1.

**Table 1:** Evaluation of the included health economic studies on endotracheal tubes in preventing ventilator-associated pneumonia (VAP) with the updated consolidated health economic evaluation reporting standards (CHEERS) statement.

Study	CHEERS items satisfied	CHEERS items not satisfied	Relevant CHEERS items	Percent (%) satisfied	Quality
Andronis et al 2018	21	4	26	81	High
Menegueti et al 2016	21	4	26	81	High
Branch-Elliman et al 2015	21	4	26	81	High
Speroni et al 2011	17	8	25	68	Medium

### 3.3. Baseline characteristics

Out of the 4 included studies, 2 investigations were undertaken in the United States,^[15,16]^ with 1 study each conducted in the United Kingdom^[17]^ and Brazil.^[18]^ All of the research protocols utilized an economic model, with the exception of 1 study which employed a comparative analysis.^[15]^

The investigations included adult patients who were admitted to the ICU for a duration ranging from 12 hours in 1 study^[16]^ to 48 hours. The chosen studies comprised a collective of 1069 patients, with a mean age of 66.8 years. Two of the conducted research studies focused solely on the hospital’s viewpoint,^[15,18]^ whilst 1 study considered both the societal and hospital perspective.^[16]^ The last remaining study utilized the perspective of the NHS healthcare system.^[17]^ Moreover, the time horizon of included studies ranged from 12 days to 23 months.

The research encompassed various cost categories, with the most prevalent being the acquisition cost and purchase cost of the ETT. Additionally, preventative costs and hospital expenses were also taken into account. Lastly, in the chosen studies, the evaluation of clinical outcomes primarily focused on the prevention of VAP. However, 1 study included incorporated the health outcomes of quality-adjusted life years (QALY).^[17]^ Table 2 displayed the baseline characteristics of included studies.

**Table 2:** Methodological characteristics of health economic studies on endotracheal tubes in preventing ventilator-associated pneumonia (VAP).

Study	Study type and country	Underlying illness	Sample demographics	Type of endotracheal tube	Cost analyzed	Duration	Perspective
Andronis et al 2018	Model based economic evaluation, UK	Patients requiring mechanical ventilation after cardiac surgery	n = 240 (120 + 120). The mean (SD) ages were 72.4 (8.2) and 72.1 (7.4) years.	Venner-PneuX (VPX) system vs standard endotracheal tube (ETT)	Acquisition cost of VPX and SET, cost of care for patients with and without VAP	28 d	NHS secondary healthcare service providers
Menegueti et al 2016	Model based cost-effectiveness, Brazil	Adult ICU patients under mechanical ventilation for over 48 h	n = 314 (168 + 146). The mean ages were 76 and 52 years.	ETT with heat and moisture exchangers (HME) filter vs ETT with heated humidifiers (HH)	Costs of HME and HH, including purchase	12 d	Hospital
Branch-Elliman et al 2015	Model based cost-benefit analysis, USA	Adult ICU patients under mechanical ventilation for over 12 h	n = 361.	Subglottic suction ETT vs silver-coated ETT	Prevention strategy costs, including device and care costs	28 d	Hospital and Societal
Speroni et al 2011	Comparative effectiveness, USA	Adult ICU patients	154 (77 + 77). The mean (SD) ages were 66.7 (14.2) and 61.6 (15.7) years.	Continuous subglottic suctioning ETT (CSS-ETT) vs standard ETT (S-ETT)	Acquisition costs, hospital charges	23 mo	Hospital perspective

ICU = intensive care unit, NHS = National Health Services.

### 3.4. Interventional characteristics of included studies

There were 4 distinct variants of ETT that were employed in the selected studies. Two studies compared the cost and clinical outcomes of Venner-PneuX (VPX) system ETT and the continuous subglottic suctioning ETT (CSS-ETT) with the standard ETT (S-ETT).^[15,17]^ The third study compared ETT equipped with heat and moisture exchangers (HME) filters to those equipped with heated humidifiers (HH).^[18]^ In a study conducted by Branch-Elliman et al in 2015, various strategies for preventing VAP were evaluated. However, the most prominent comparison was between the use of subglottic suction ETT and silver-coated ETT. The following details report on each ETT that were concluded as cost-effective in the respective studies.

#### 3.4.1. Venner-PneuX endotracheal tube system

A decision analytic model was developed to evaluate the cost-effectiveness of Venner-PneuX endotracheal tube system in comparison to the standard ETT from a healthcare perspective. Venner-PneuX (VPX; Venner Group of Companies) is an endotracheal system designed to monitor, regulate, and uphold a secure inflation volume and pressure (30 cm H_2_O) within the cuff.

The study found that the Venner-PneuX endotracheal tube system is a cost-effective measure for avoiding VAP in patients who have undergone cardiac surgery. It was regard as superior option to standard ETT, offering both economic and therapeutic benefits. The Venner-PneuX system is highly likely to be cost-effective, with a 97% likelihood, when considering a willingness-to-pay threshold of £30,000 per QALY. The system provides a decrease in direct healthcare expenses, particularly by reducing the occurrence of VAP (10.8% vs 21%) and shortening stays in the ICU. Additionally, it contributes to enhancing patient overall health outcomes, as shown by a modest rise in QALYs. Similarly, from the standpoint of the healthcare provider, substantial cost reductions, estimated at £738 per patient was observed.^[17]^

#### 3.4.2. Endotracheal tube with HME filters

The Menegueti et al 2016 assesses the cost-effectiveness of using a decision tree model to compare the use of ETT with HME and ETT with HH in reducing VAP among ICU mechanically ventilated patients. The research findings clearly support the use of HMEs, since they are shown to be a more economically feasible choice with a smaller cost burden, while yet being equally efficient in preventing VAP. The study delineates the precise economic benefits of HMEs, encompassing their lower initial acquisition costs and diminished ongoing operational expenses, which together enhance their total cost-effectiveness. Moreover, the therapeutic advantages of utilizing HMEs, such as their comparability in decreasing the occurrence of VAP to that of HHs, reinforce its use. In particular, the cost of HME varied between R$ 21,000.00 and R$ 22,000.00, while the effectiveness for preventing VAP was approximately 82.6%. In comparison, the cost of HH for achieving an effectiveness of 81.3% in preventing VAP was R$ 30,000.00.^[18]^

#### 3.4.3. Subglottic suction ETT

Elliman et al 2015, did a thorough investigation on the cost-effectiveness of different strategies for preventing VAP. They highlighted the economic and therapeutic benefits of these strategies from both hospital and societal perspective. For this purpose, an economic Markov model was developed. The study emphasizes the effectiveness of using subglottic suction ETT, as opposed to standard and silver-coated ETT. The subglottic suction ETT not only shows a reduction in VAP rates, but also leads to substantial cost savings by reducing the number of days’ patients require mechanical ventilation and shortening their stays in the ICU. In particular, the Subglottic suction ETT had a cost of $17.16 and resulted in a risk reduction of 0.61 for VAP. On the other hand, the silver-coated ETT had a cost of $50 and resulted in a risk reduction of 0.51 for VAP. In conclusion, the study underscores the limited efficacy of silver-coated endotracheal tubes, and advocated for the cost-effectiveness and clinical advantages of alternate approaches including subglottic suction ETT.^[16]^

#### 3.4.4. Continuous subglottic suctioning endotracheal tubes

In contrast to the previous 3 studies that employed an economic model, the study conducted by Speroni et al 2011 undertakes a comparative analysis. The aim of this study was to assess the relative costs of CSS-ETT compared to S-ETT in intubated patients, and to determine if the cost difference is balanced out by the occurrence of VAP in patients receiving either type of intubation. A retrospective analysis of medical records was performed for a total of 154 adult patients who were intubated. Among them, 77 patients were intubated using a S-ETT and the remaining 77 patients were intubated using a CSS-ETT. The findings showed that the S-ETT group experienced 1 case of VAP, while the CSS-ETT group did not have any cases. Similarly, the average total hospital charges were greater for the S-ETT group ($103,600 vs $88,500). Therefore, CSS-ETT was concluded as cost-effective in comparison to S-ETT as per hospital perspective.^[15]^ The interventional summary of included studies is summarized in Table 3.

**Table 3:** Interventional characteristics in the included health economic studies on endotracheal tubes in preventing ventilator-associated pneumonia (VAP).

Study	Experimental			Control			Economic outcome	Reported cost year	Conclusion
	Type	Net cost	Net outcome	Type	Net cost	Net outcome			
Andronis et al 2018	Venner-PneuX (VPX) system ETT	£7401	0.025 QALY and 10.8% infection rate	Standard ETT (S-ETT)	£8139	0.024 QALY and 21% infection rate	Cost saving of £738 per patient for VPX	2016	VPX cost-effective
Menegueti et al 2016	ETT with heat and moisture exchangers (HME) filter	R$ 21,552.52	82.6% effectiveness and 8.6% infection rate	ETT with heated humidifiers (HH)	R$ 30,418.08	81.3% effectiveness and 9.24% infection rate	ICER: R$ 681,966.16 for HME vs HH	2011	Though very little difference in effectiveness but HME is more advantageous
Branch-Elliman et al 2015	Subglottic suction ETT	$17.16 ($10–100)	0.61 VAP risk reduction	Silver-coated ETT	$50 ($30–60)	0.51 VAP risk reduction	Subglottic suction ETT dominant	2014	Subglottic suction ETT cost-effective
Speroni et al 2011	Continuous subglottic suctioning ETT (CSS-ETT)	$88,498	0.013% infection rate	S-ETT	$103,594	0% infection rate	CSS-ETT dominant	2007	CSS-ETT cost-effective

ETT = endotracheal tubes, NR = not reported.

## 4. Discussion

The aim of this systematic analysis was to comprehensively assess the health economic implications of several modifications of ETT in the prevention of VAP. While there are existing systematic reviews that have evaluated the clinical efficacy of these ETT in preventing VAP, there is currently no evaluation available regarding their cost-effectiveness. Thus, to our knowledge, this is the initial evaluation that examined the cost-effectiveness of ETT in preventing VAP. The combined results of the included studies suggest that modifications of the ETT is both useful in a clinical setting and cost-efficient when compared to the standard ETT, from both a societal and hospital perspective. However, due to the limited number of studies and the emphasis on different types of ETT, intra-comparison of these modified ETTs was not possible.

The Venner-PneuX endotracheal tube system stands apart from other ETTs due to its use of several strategies to minimize the intake of oropharyngeal secretions. For instance, these possess subglottic suction apertures which are utilized for the removal of accumulated waste in the subglottic region. In these ETTs, the cuff with low pressure and volume can inflate even in the absence of creases or folds, while the tracheal seal monitor ensures optimal cuff pressure is maintained. Furthermore, these contain a nonstick covering that prevents the adherence of microorganisms and the formation of biofilms.^[19]^ Due to these reasons, previous research acknowledged the clinical efficacy of these ETTs in prevention of VAP.^[19,20]^ However, relying on a single study is insufficient to conclusively determine their cost-effectiveness^[17]^ and further investigation, particularly through randomized controlled trials, is warranted. This approach is also applicable to the ETT with HME filter, which was included in only a single study and was found cost-effective in comparison to ETT with HH filter only.^[18]^

There were 2 studies that concluded the cost-effectiveness of subglottic suction ETTs. Branch-Elliman et al utilized an economic model and analyzed 120 strategies, including both ETTs and non-ETTs techniques for the prevention of VAP.^[21]^ Similarly, Spironi et al who made a comparative analysis of the CSS-ETT with standard ETT. As VAP is predominantly caused by the inhalation of infectious debris generated by the oropharyngeal region.^[22]^ Thus, it may be inferred that the use of an ETT with subglottic suction technique may be both economically advantageous and clinically effective.^[15,17,21]^

While prior studies have presented evidence supporting the clinical and cost-effectiveness of silver-coated ETTs.^[23,24]^ However, none of the included studies in the analysis found evidence supporting the high cost-effectiveness of silver-coated ETTs. Nonetheless, it is crucial to acknowledge that the prior research solely assessed the efficacy of silver-coated ETTs without doing comparisons with alternative preventive strategies, resulting in varying outcomes. Furthermore, with the advancement of technology, new forms of ETTs are appearing, making the previous findings obsolete.

In summary, the studies analyzed in our review present convincing evidence that advanced ETT designs, such as those with subglottic secretions, not only reduce the occurrence of VAP but also yield significant economic advantages. Our research indicates that the economic efficiency of ETT advances justifies their inclusion into clinical practice. However, decision-makers should also take into account variables such as the practicality of implementation, training of personnel, and the possible requirement for modifications in clinical protocols.

There are certain limitations of the systematic review that cannot be ignored. For instance, the research included in the analysis were conducted solely in specific countries, including the United States, United Kingdom, and Brazil. No pertinent economic data pertaining to low and middle-income countries was identified. Hence, it is crucial to acknowledge that the findings may not be applicable to countries with dissimilar economic conditions, owing to the variations in economic frameworks among nations. Furthermore, while there was consistency in assessing the health outcomes associated with VAP, the studies differed in their approaches for types of cost, perspective, duration, and the type of ETT utilized. Similarly, the cost-effectiveness of a particular intervention, is dependent upon the willingness-to-pay, but, in our case, no individual study compares the economic outcome with the willingness-to-pay of involved country.

In the future, it is important to perform more extensive economic assessments by utilizing larger and more representative sample sizes. Furthermore, it is crucial to conduct economic evaluations in numerous countries, particularly in low and middle-income nations, in order to improve the applicability of the findings. Lastly, future studies must use a comprehensive strategy when assessing the economic consequences of medical interventions of ETT for the prevention of VAP. This encompasses not only the cost linked to the acquisition of ETT but also the wider financial consequences, such as hospital and intensive care unit stays, and the impact on indirect medical costs.

## 5. Conclusion

The studies analyzed in our review present convincing evidence that advanced ETT designs, such as those with subglottic secretions, not only reduce the occurrence of VAP but also yield significant economic advantages. Our research indicates that the economic efficiency of ETT advances justifies their inclusion into clinical practice. However, decision-makers should also take into account variables such as the practicality of implementation, training of personnel, and the possible requirement for modifications in clinical protocols.

## Author contributions

**Conceptualization:** Safeer Khan.

**Data curation:** Benjamin De Vlam.

**Methodology:** Safeer Khan.

**Software:** Guy Hans.

**Supervision:** Safeer Khan.

**Validation:** Benjamin De Vlam.

**Writing – original draft:** Benjamin De Vlam, Guy Hans.

**Writing – review & editing:** Safeer Khan.

## Supplementary Material


