# Supplementary material for: A systematic review of health economic studies on endotracheal tubes in preventing ventilator-associated pneumonia
Source: Medicine (Baltimore). 2025 Aug 8;104(32):e43877. doi: 10.1097/MD.0000000000043877 (PMC12338191; doi:10.1097/MD.0000000000043877)
Supplement: Supplementary file 1 [file medi-104-e43877-s001.docx]

**Table:** Search Strategy

| **Keywords** | **Database/Search Engine** | **No. of retrieved results** |
| --- | --- | --- |
| (("Costs and Cost Analysis"[MeSH Terms] OR "Cost-Benefit Analysis"[MeSH Terms] OR "Cost-Effectiveness Analysis"[MeSH Terms] OR "Health Care Costs"[MeSH Terms]) AND ("Airway Extubation"[MeSH Terms] OR "intubation, intratracheal"[MeSH Terms] OR "anesthesia, endotracheal"[MeSH Terms])) AND (2010:2024[pdat]) | PubMed | 112 |
| (("economics"[MeSH Subheading] OR "economics"[All Fields] OR "cost"[All Fields] OR "costs and cost analysis"[MeSH Terms] OR ("costs"[All Fields] AND "cost"[All Fields] AND "analysis"[All Fields]) OR "costs and cost analysis"[All Fields]) AND (("endotracheal"[All Fields] OR "endotracheally"[All Fields]) AND ("tube s"[All Fields] OR "tubed"[All Fields] OR "tubes"[All Fields] OR "tubing"[All Fields] OR "tubings"[All Fields])) AND ("pneumonia, ventilator associated"[MeSH Terms] OR ("pneumonia"[All Fields] AND "ventilator associated"[All Fields]) OR "ventilator-associated pneumonia"[All Fields] OR ("ventilator"[All Fields] AND "associated"[All Fields] AND "pneumonia"[All Fields]) OR "ventilator associated pneumonia"[All Fields])) AND (2010:2024[pdat]) | PubMed | 16 |
| (("economical"[All Fields] OR "economics"[MeSH Terms] OR "economics"[All Fields] OR "economic"[All Fields] OR "economically"[All Fields] OR "economics"[MeSH Subheading] OR "economization"[All Fields] OR "economize"[All Fields] OR "economized"[All Fields] OR "economizes"[All Fields] OR "economizing"[All Fields]) AND ("impact"[All Fields] OR "impactful"[All Fields] OR "impacting"[All Fields] OR "impacts"[All Fields] OR "tooth, impacted"[MeSH Terms] OR ("tooth"[All Fields] AND "impacted"[All Fields]) OR "impacted tooth"[All Fields] OR "impacted"[All Fields]) AND (("endotracheal"[All Fields] OR "endotracheally"[All Fields]) AND ("tube s"[All Fields] OR "tubed"[All Fields] OR "tubes"[All Fields] OR "tubing"[All Fields] OR "tubings"[All Fields]))) AND (2010:2024[pdat]) | PubMed | 3 |
| (("cost effectiveness analysis"[MeSH Terms] OR ("cost effectiveness"[All Fields] AND "analysis"[All Fields]) OR "cost effectiveness analysis"[All Fields] OR ("cost"[All Fields] AND "effectiveness"[All Fields]) OR "cost effectiveness"[All Fields]) AND ("VAP"[All Fields] AND ("prevent"[All Fields] OR "preventability"[All Fields] OR "preventable"[All Fields] OR "preventative"[All Fields] OR "preventatively"[All Fields] OR "preventatives"[All Fields] OR "prevented"[All Fields] OR "preventing"[All Fields] OR "prevention and control"[MeSH Subheading] OR ("prevention"[All Fields] AND "control"[All Fields]) OR "prevention and control"[All Fields] OR "prevention"[All Fields] OR "prevention s"[All Fields] OR "preventions"[All Fields] OR "preventive"[All Fields] OR "preventively"[All Fields] OR "preventives"[All Fields] OR "prevents"[All Fields]))) AND (2010:2024[pdat]) | PubMed | 30 |
| (("cost effectiveness analysis"[MeSH Terms] OR ("cost effectiveness"[All Fields] AND "analysis"[All Fields]) OR "cost effectiveness analysis"[All Fields] OR ("cost"[All Fields] AND "effectiveness"[All Fields]) OR "cost effectiveness"[All Fields]) AND ("VAP"[All Fields] AND ("prevent"[All Fields] OR "preventability"[All Fields] OR "preventable"[All Fields] OR "preventative"[All Fields] OR "preventatively"[All Fields] OR "preventatives"[All Fields] OR "prevented"[All Fields] OR "preventing"[All Fields] OR "prevention and control"[MeSH Subheading] OR ("prevention"[All Fields] AND "control"[All Fields]) OR "prevention and control"[All Fields] OR "prevention"[All Fields] OR "prevention s"[All Fields] OR "preventions"[All Fields] OR "preventive"[All Fields] OR "preventively"[All Fields] OR "preventives"[All Fields] OR "prevents"[All Fields]))) AND (2010:2024[pdat]) | PubMed | 30 |
| (("economics"[MeSH Subheading] OR "economics"[All Fields] OR "cost"[All Fields] OR "costs and cost analysis"[MeSH Terms] OR ("costs"[All Fields] AND "cost"[All Fields] AND "analysis"[All Fields]) OR "costs and cost analysis"[All Fields]) AND (("intubate"[All Fields] OR "intubated"[All Fields] OR "intubates"[All Fields] OR "intubating"[All Fields] OR "intubation"[MeSH Terms] OR "intubation"[All Fields] OR "intubations"[All Fields] OR "intubator"[All Fields] OR "intubator s"[All Fields] OR "intubators"[All Fields]) AND ("tube s"[All Fields] OR "tubed"[All Fields] OR "tubes"[All Fields] OR "tubing"[All Fields] OR "tubings"[All Fields])) AND ("pneumonia, ventilator associated"[MeSH Terms] OR ("pneumonia"[All Fields] AND "ventilator associated"[All Fields]) OR "ventilator-associated pneumonia"[All Fields] OR ("ventilator"[All Fields] AND "associated"[All Fields] AND "pneumonia"[All Fields]) OR "ventilator associated pneumonia"[All Fields])) AND (2010:2024[pdat]) | PubMed | 15 |
| ("Cost"[All Fields] AND "Coatings"[All Fields] AND "Endotracheal tubes"[All Fields]) AND (2010:2024[pdat]) | PubMed | 1 |
| ("Cost"[All Fields] AND "Prevention"[All Fields] AND "ventilator-associated pneumonia"[All Fields] AND "Intubation"[All Fields]) AND (2010:2024[pdat]) | PubMed | 29 |
| ("Cost-Effectiveness"[All Fields] AND "VAP Prevention"[All Fields]) AND (2010:2024[pdat]) | PubMed | 6 |
| ("Cost Analysis"[All Fields] AND "Endotracheal tubes"[All Fields]) AND (2010:2024[pdat]) | PubMed | 1 |
| Year: 2013-2024 Title, abstract, keywords: Ventilator-Associated Pneumonia AND Cost AND Endotracheal tubes | ScienceDirect | 2 |
| Year: 2013-2024 Title, abstract, keywords: Ventilator-Associated Pneumonia AND economic impact AND Endotracheal Tubes | ScienceDirect | 1 |
| Year: 2013-2024 Title, abstract, keywords: Ventilator-Associated Pneumonia AND economic AND Antimicrobial Coatings in Endotracheal tubes | ScienceDirect | 2 |
| Year: 2013-2024 Title, abstract, keywords: Cost-Effectiveness of VAP Prevention | ScienceDirect | 4 |
| Year: 2013-2024 Title, abstract, keywords: Cost-Effectiveness of Ventilator-Associated Pneumonia Prevention | ScienceDirect | 6 |
| Year: 2013-2024 Title, abstract, keywords: Cost of Ventilator-Associated Pneumonia Prevention | ScienceDirect | 21 |
| Year: 2013-2024 Title, abstract, keywords: Cost AND Ventilator Associated Pneumonia Prevention | ScienceDirect | 21 |
| Year: 2013-2024 Title, abstract, keywords: Cost of ETT tubes | ScienceDirect | 2 |
| Year: 2013-2024 Title, abstract, keywords: Cost of Intubation Tubes | ScienceDirect | 15 |
| Year: 2013-2024 Title, abstract, keywords: Cost AND Intubation Tubes AND Ventilator-Associated Pneumonia | ScienceDirect | 1 |
| Year: 2013-2024 Title, abstract, keywords: Cost of ETT tubes | ScienceDirect | 2 |
| allintitle: Cost and Prevention AND Ventilator-Associated Pneumonia | Google Scholar | 1 |
| allintitle: Cost and Prevention AND Endotracheal tubes | Google Scholar | 1 |
| allintitle: Cost AND Respiratory tubes | Google Scholar | 1 |
| allintitle: Cost-Effectiveness of VAP Prevention | Google Scholar | 1 |
| Cost of Endotracheal Tubes | HTA | 1 |
| Cost of ET tubes | HTA | 5 |
| Cost AND Endotracheal tubes | CEA | 2 |
| Cost AND Intubation | CEA | 10 |
| Endotracheal Tube | CEA | 1 |
